# Actionability evaluation of biliary tract cancer by genome transcriptome analysis and Asian cancer knowledgebase

**DOI:** 10.18632/oncotarget.28021

**Published:** 2021-07-20

**Authors:** Yuki Okawa, Nobutaka Ebata, Nayoung K.D. Kim, Masashi Fujita, Kazuhiro Maejima, Shota Sasagawa, Toru Nakamura, Woong-Yang Park, Satoshi Hirano, Hidewaki Nakagawa

**Affiliations:** ^1^Laboratory for Cancer Genomics, RIKEN Center for Integrative Medical Sciences, Yokohama, Japan; ^2^Department of Gastroenterological Surgery II, Hokkaido University Faculty of Medicine, Sapporo, Japan; ^3^Geninus Inc., Seoul, Republic of Korea; ^4^Samsung Genome Institute, Samsung Medical Center, Seoul, Republic of Korea

**Keywords:** biliary tract cancer, mutation, immunotherapic biomarker, knowledgebase, molecular targeted therapy

## Abstract

Introduction: Treatment options for biliary tract cancer (BTC) are very limited. It is necessary to investigate actionable genes and candidate drugs using a sophisticated knowledgebase (KB) and characterize BTCs immunologically for evaluating the actionability of molecular and immune therapies.

Materials and Methods: The genomic and transcriptome data of 219 patients with BTC who underwent surgery were analyzed. Actionable mutations and candidate drugs were annotated using the largest available KB of the Asian population (CancerSCAN^®^). Predictive biomarkers of immune checkpoint inhibitors were analyzed using DNA and RNA sequencing data.

Results: Twenty-two actionable genes and 43 candidate drugs were annotated in 74 patients (33.8%). The most frequent actionable genes were *PTEN* (7.3%), *CDKN2A* (6.8%), *KRAS* (6.4%). *BRCA2*, *CDKN2A*, and *FGFR2* mutations were most frequently identified in case of intrahepatic cholangiocarcinoma. *PTEN* and *CDKN2A* mutations were associated with significantly shorter overall survival. *PD-L1* and *PD-1* expression was significantly higher in case of extrahepatic cholangiocarcinoma and T-cell-high expression. In total, 49.7% of cases were evaluated as having actionability for molecular therapy or immune checkpoint inhibitors.

Conclusions: Identifying actionable genes and candidate drugs using the KB contribute to the development of therapeutic drugs and personalized treatment for BTC.

## INTRODUCTION

Biliary tract cancer (BTC) or cholangiocarcinoma, which originates from bile duct epithelial cells (cholangiocytes), is a rare tumor worldwide. However, it is prevalent in some areas with a high incidence of specific risk factors such as chronic inflammation of the biliary tract and gallbladder and hepatitis, as well as chemical exposure such as aflatoxin [1, 2]. According to their diverse anatomical locations, BTCs are mainly classified as intrahepatic (ICC) or extrahepatic cholangiocarcinoma (ECC), including the peri-hilar type (PHC or Klatskin tumor), or gallbladder cancer (GBC). Regardless of their anatomical location or pathology, BTCs are very aggressive, with high metastatic and invasive potential, and are difficult to completely resect by surgery because of their complicated anatomical location and spread along the bile ducts [3]. Although the first line of treatment is surgical resection, the relapse rate is 50–60% and the 5-year survival rate is only 30% even after achieving R0 resection [4]. No adjuvant therapies with established therapeutic effects are available for BTC and gemcitabine-based chemotherapy is a standard regimen for relapse BTCs [5].

The genetic features of BTCs remain poorly understood because their molecular profiles are as heterogeneous as their pathology and biology. Several studies on the genomic alterations in various BTC types have commonalities, such as *TP53*, *KRAS*, *SMAD4*, *ARID1A*, *CDKN2A*, *IDH1*, and *ELF3* mutations [6–8]. However, most of these are not “actionable” genes or mutations, and there are only few actionable genes or mutations that can be targeted specifically in BTC by any molecular therapy or drug. The European Society for Medical Oncology (ESMO) recommends genetic alterations, such as mutations in *IDH1* and *FGFR2* fusion, as molecular targets of clinical actionability in BTC [9]. Clinical trials have also been conducted in advanced cholangiocarcinoma with *IDH1* mutations [10] and *FGFR2* fusions [11]. However, these are still in the research phase, and there are no approved molecularly targeted therapies available. The lack of newly approved drugs is due to the rarity of BTC and its pathological and molecular heterogeneity, which makes it challenging to conduct phase III randomized controlled trials [5].

In addition, the adaptation of immune checkpoint inhibitors (ICIs) has expanded in recent years for almost all cancer types [12]. Microsatellite instability (MSI) status, tumor-infiltrating lymphocytes, tumor mutation burden (TMB), and *PD-L1* expression have been investigated as candidate biomarkers for ICIs [13]. However, in BTC, TMB-High is found in approximately 25% of cases, whereas the frequency of MSI-high is less than 3% [14]. Efficacy of the *PD-1* inhibitor pembrolizumab was observed in advanced BTC regardless of *PD-L1* CPS-positive status [15], whereas higher *PD-L1* expression in BTC was associated with the response rate of pembrolizumab [16]. Overall, the biomarkers of ICI in BTC are still unknown, and their analysis is urgently needed.

Regarding the clinical annotations of cancer genome variants, several knowledge bases (KBs) integrating massive amounts of genomic and clinical information data from articles and clinical trials have been developed to identify actionable genes and candidate drugs [17, 18]. Detection of known actionable genes and accumulation of data using these KBs may promote the adaptation and development of new therapeutic agents and clinical trials for them.

This study aimed to detect actionable genes or mutations and candidate drugs in BTC using a KB with research-level information on genomic mutation lists and to investigate the relationship between actionable genes and clinic-pathological features. We also analyzed TMB, T cell expression, and *PD-L1* and *PD-1* expression, which are candidate biomarkers for ICI, to reveal the immunological characteristics and immunological actionability in BTC.

## RESULTS

### Annotation of actionable mutations

We accumulated the data of WGS, WES, targeted sequencing, and RNA-seq from BTCs [19] and ICCs [20, 21]. In this study, we re-analyzed these genomic data after including the data from one sample and annotated the detected mutations and aberrant expressions in 219 BTC cases in total. Their clinicopathological characteristics are shown in Supplementary Table 1 and summarized in Table 1. The median age was 69 (44–86) years, and 27.4% (*n* = 60) were female. By the BTC location, ICC was the most common (30.1% [*n* = 66]), followed by perihilar cholangiocarcinoma (PHC), distal cholangiocarcinoma (DCC), and gallbladder carcinoma (GBC), including cystic ductal carcinoma (CDC). The most common pathological staging was stage III (36.1% [*n* = 79]), and 35.2% (*n* = 77) had lymph node metastases.

**Table 1:** Characteristics of 219 patients with biliary tract cancer

	Patients (*n* = 219)	
**Age, years**		
Median (range)	69	(44–86)
Sex		
Male	159	(72.6)
Female	60	(27.4)
**The location of BTC**		
ICC	66	(30.1)
PHC	63	(28.8)
GBC/CDC	41	(18.7)
DCC	49	(22.4)
**Pathological lymph node metastasis**		
(–)	138	(63)
(+)	77	(35.2)
NA	4	(1.8)
**Pathological Stage according to AJCC/UICC 7th**		
I	29	(13.2)
II	59	(26.9)
III	79	(36.1)
IV	49	(22.4)
NA	3	(1.4)
**Histology**		
Tubular adenocarcinoma	178	(81.3)
Poorly differentiated adenocarcinoma	27	(12.3)
Adenosquamous adenocarcinoma	5	(2.3)
Mucinous adenocarcinoma	2	(0.9)
Others	3	(1.4)
NA	4	(1.8)

Data are expressed as number (%) or median (minimum value - maximum value). Abbreviations: BTC: biliary tract cancer; ICC: intrahepatic cholangiocarcinoma; PHC: perihilar cholangiocarcinoma; GBC: gallbladder carcinoma; CDC: cystic duct carcinoma; DCC: distal cholangiocarcinoma; NA: not available.

We annotated the actionable mutations using a KB containing the frequency data of approximately 15,000 East Asians as well as drug mapping information. Using this KB, we analyzed 369 genes for SNVs, indels, and CNVs using genomic DNA and the fusions of 627 genes using capture-based RNA-seq. The list of genes used in the analysis is listed in Supplementary Tables 2 and 3. WGS, WES, and targeted sequencing revealed 602 SNVs and 28 indels in protein-coding regions and splice sites, respectively, and 512 non-coding mutations. We annotated 41 SNVs and one indel using the KB for actionability (Supplementary Table 4). WGS revealed 392 CNVs and we annotated 33 CNVs; WES revealed 635 CNVs of which we annotated 46 CNVs (Supplementary Table 5). RNA-seq revealed 51 fusions and of which 15 fusions were annotated (Supplementary Table 6).

**Figure 1:**
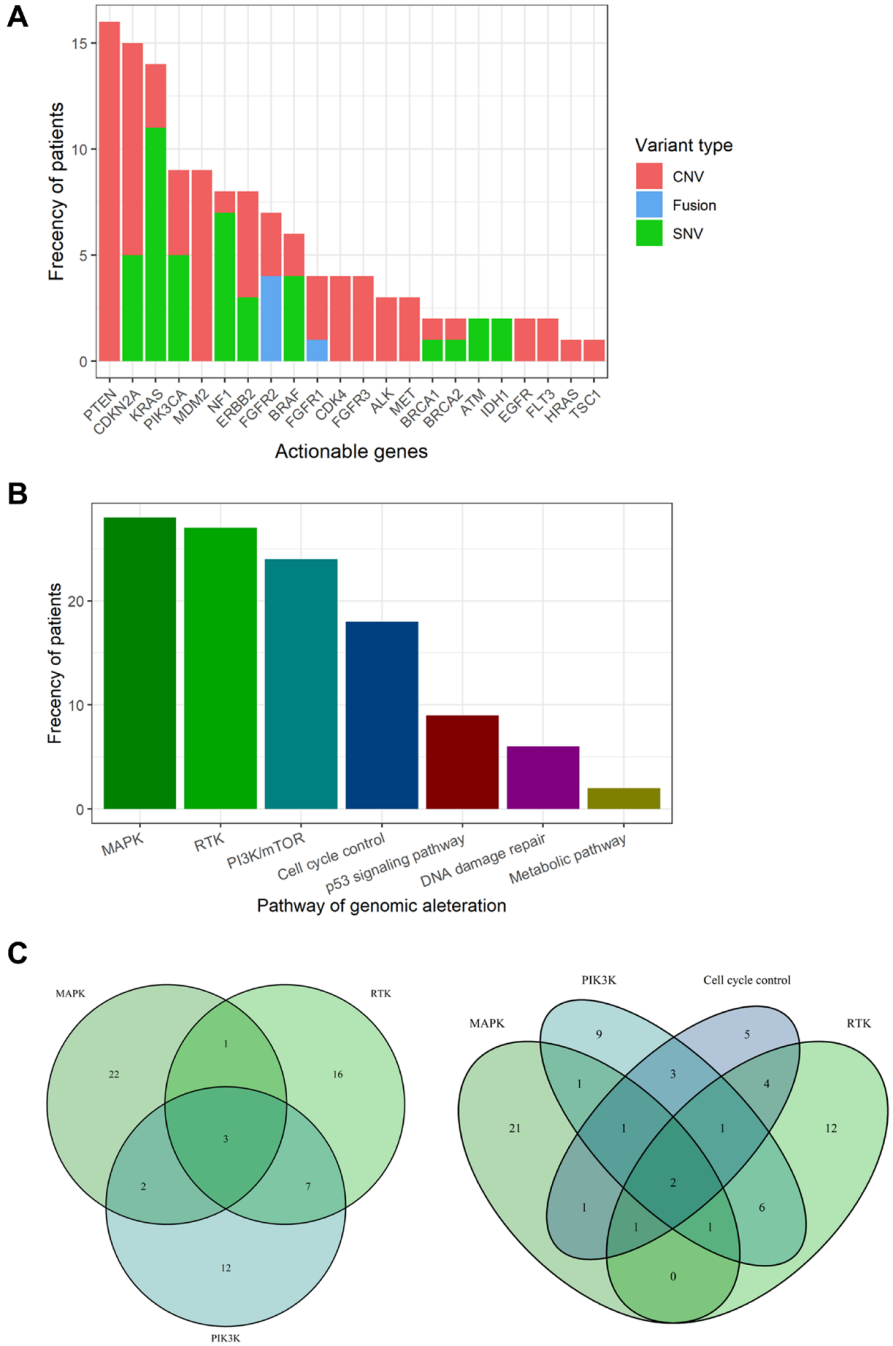
Actionable genes and pathways in biliary tract cancer. (**A**) Bar plot showing the frequency of actionable genes in biliary tract cancer. (**B**) Bar plot showing the frequency of actionable pathways in biliary tract cancer. (**C**) Venn diagram showing patients with overlapping actionable pathways. Venn diagrams for the top three pathways (left) and the top four pathways (right).

Aggregating these results, 22 actionable genes and 43 candidate drugs were annotated in 74 cases using the KB (Figure 1A and Supplementary Table 7). The most frequent actionable genes were *PTEN* (7.3%), followed by *CDKN2A* (6.8%), *KRAS* (6.4%), *PIK3CA* (4.1%), and *MDM2* (4.1%). All variants of *PTEN* showed copy number loss (Supplementary Table 5). Only *FGFR1/2* were found as the actionable gene caused by fusion.

Actionable genes were aggregated in the following pathways (Figure 1B): MAPK pathway (12.8%), RTK pathways (12.3%), PI3K pathway (11%), cell cycle control (8.2%), p53 signaling pathway (4.1%), DNA damage repair (2.7%), and metabolic pathways (0.9%). Three cases showed duplication of the top three actionable pathways, and two cases showed duplication of the top four actionable pathways (Figure 1C). One case (RK138) was found to have up to seven actionable genes (*ALK, CDK4, CDKN2A, KRAS, MET, NF1,* and *PIK3CA*). In total, 33.8% of the BTC cases had at least one actionable mutation.

### Comparison between actionable mutations and clinicopathological features

Next, the relationship between actionable mutations and clinicopathological features in BTC was investigated. Based on the anatomical location of BTC in pathological diagnosis [22], PHC, GBC/CDC, and DCC were integrated as ECC, and actionable mutations were compared for ICC and ECC (Table 2). As a result, actionable mutations were more frequently identified in ICC (51.5%, 33 of 66 patients) (*P* = 0.001). *BRCA2, CDKN2A,* and *FGFR2* mutations were most frequently identified in ICC (*P* = 0.03, *P* = 0.02, and *P* = 0.003, respectively). In contrast, 73.2% of ECCs were found to have no actionable mutations. Pathological lymph node metastasis and sex were examined, and no significant differences in actionable genes were identified (Supplementary Tables 8 and 9).

**Table 2:** Comparison between actionable genes and the location of biliary tract cancer

Actionable gene	ICC *n* = 66		ECC *n* = 153		*P*-value	OR	95% CI
Actionability (+)	33	(50)	41	(26.8)	0.001	2.73	(1.5–4.98)
*ALK*	2	(3)	1	(0.7)	0.22	4.71	(0.36–138.58)
*ATM*	0	(0)	2	(1.3)	1	0	(0–8.06)
*BRAF*	3	(4.5)	3	(2)	0.37	2.37	(0.42–13.44)
*BRCA1*	1	(1.5)	1	(0.7)	0.51	2.33	(0.06–90.58)
*BRCA2*	3	(4.5)	0	(0)	0.03	Inf	(1.38-Inf)
*CDK4*	3	(4.5)	1	(0.7)	0.08	7.17	(0.78–188.1)
*CDKN2A*	9	(13.6)	6	(3.9)	0.02	3.84	(1.29–11.43)
*EGFR*	0	(0)	2	(1.3)	1	0	(0–8.06)
*ERBB2*	2	(3)	6	(3.9)	1	0.77	(0.11–4.27)
*FGFR1*	2	(3)	2	(1.3)	0.59	2.35	(0.25–22.13)
*FGFR2*	6	(9.1)	1	(0.7)	0	15.01	(2.03–346.55)
*FGFR3*	1	(1.5)	3	(2)	1	0.77	(0.03–7.12)
*FLT3*	2	(3)	0	(0)	0.09	Inf	(0.67- Inf)
*HRAS*	0	(0)	1	(0.7)	1	0	(0–44.05)
*IDH1*	2	(3)	0	(0)	0.09	Inf	(0.67-Inf)
*KRAS*	6	(9.1)	8	(5.2)	0.37	1.81	(0.59–5.63)
*MDM2*	5	(7.6)	4	(2.6)	0.13	3.04	(0.79–12.35)
*MET*	2	(3)	1	(0.7)	0.22	4.71	(0.36–138.58)
*NF1*	2	(3)	6	(3.9)	1	0.77	(0.11–4.27)
*PIK3CA*	4	(6.1)	5	(3.3)	0.46	1.9	(0.47–7.32)
*PTEN*	8	(12.1)	8	(5.2)	0.09	2.49	(0.88–7.05)
*TSC1*	0	(0)	1	(0.7)	1	0	(0–44.05)

Data are expressed as number (%). Statistical tests were performed using the chi-square test for the presence of Actionability and the Fisher test for each gene; *P* < 0.05 is considered statistically significant. Abbreviations: ICC: intrahepatic cholangiocarcinoma; ECC: extrahepatic cholangiocarcinoma; OR: odd ratio; CI: confidential interval.

**Figure 2:**
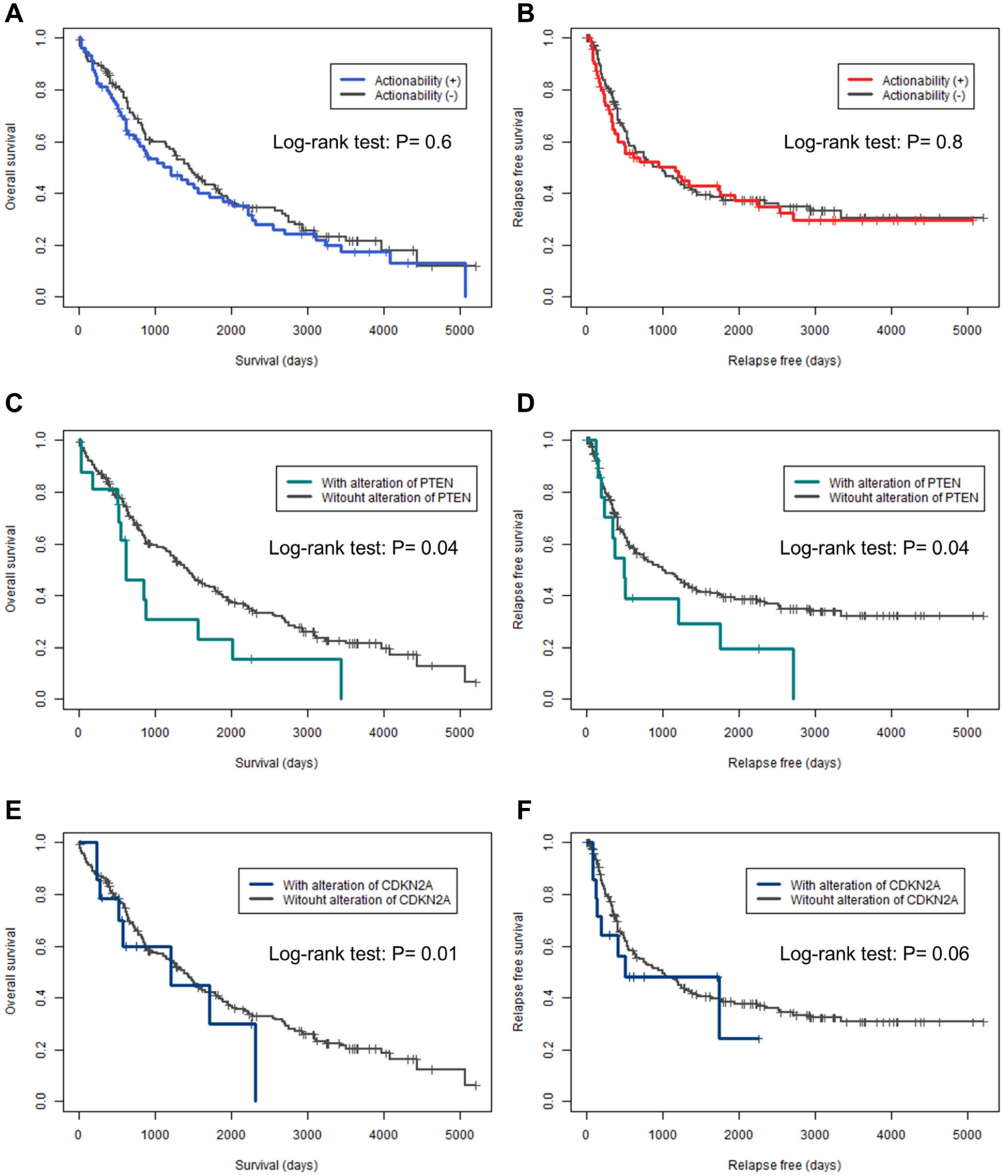
Kaplan–Meier curves for overall and relapse-free survival in actionable genes. (**A**, **C**, **E**) Kaplan–Meier curves for overall survival. (**B**, **D**, **F**) Kaplan–Meier curves for relapse-free survival. (A, B) Actionability. (C, D) *PTEN*. (E, F) *CDKN2A*. The differences in overall survival and relapse-free survival were analyzed by log-rank test; *P* < 0.05 was considered statistically significant.

Prognostic data were available for overall survival (OS) in 95% (*n* = 208) of the cases and for relapse-free survival (RFS) in 94% (*n* = 206) of the cases. Kaplan–Meier curves for OS and RFS were plotted for actionable genes with mutations in more than five cases and were analyzed using the log-rank test (Figure 2 and Supplementary Figures 1 and 2). None of the patients in this study received any candidate drugs. There was no significant difference between OS and RFS in the presence or absence of actionable genes (*P* = 0.6, and *P* = 0.8, respectively) (Figure 2A and 2B). Patients with *PTEN* mutations had significantly shorter OS and RFS (*P* = 0.04, and *P* = 0.04, respectively) (Figure 2C and 2D). For patients with *CDKN2A* mutations, the OS was significantly shorter (*P* = 0.01) and RFS tended to be shorter (*P* = 0.06). No other genetic alterations were found to shorten or prolong the OS and RFS.

### Tumor mutation burden

TMB was calculated and used as a predictive biomarker for ICI. Of the 147 patients for which WGS and WES were performed, six cases (4%) were classified as TMB-High (Table 3). The TMB of these six cases ranged from 11.1 to 58.2 mutations /Mb, and four cases were ICC. Three patients had actionable genes, whereas the others did not. Of the 72 cases with targeted sequencing performed, 18 (25%) were classified as TMB-High (Supplementary Table 10). In aggregate, 22 cases (10%) were classified as TMB-High.

**Table 3:** Characteristics of patients with TMB ≥ 10 mutations/Mbp for WGS/WES

ID	Tumor Location	Sex	Age	Number of SNV	Number of Indel	TMB	Actionable genes
RK308	ICC	F	70	2268	0	58.2	*FGFR2, PIK3CA*
RK360	GBC	F	82	1821	3	46.8	*ERBB2, NF1* *PIK3CA, CDKN2A*
HK67	DCC	M	78	771	15	35	None
HK08	ICC	M	50	807	7	20.9	*ERBB2*
HK101	ICC	M	73	1325	39	20.2	None
HK15	ICC	M	71	426	6	11.1	None

Abbreviations: BTC: biliary tract cancer; SNV: single nucleotide variant; TMB: tumor mutation burden; ICC: intrahepatic cholangiocarcinoma; GBC: gallbladder carcinoma; DCC: distal cholangiocarcinoma.

### Immune-signature analysis

**Figure 3:**
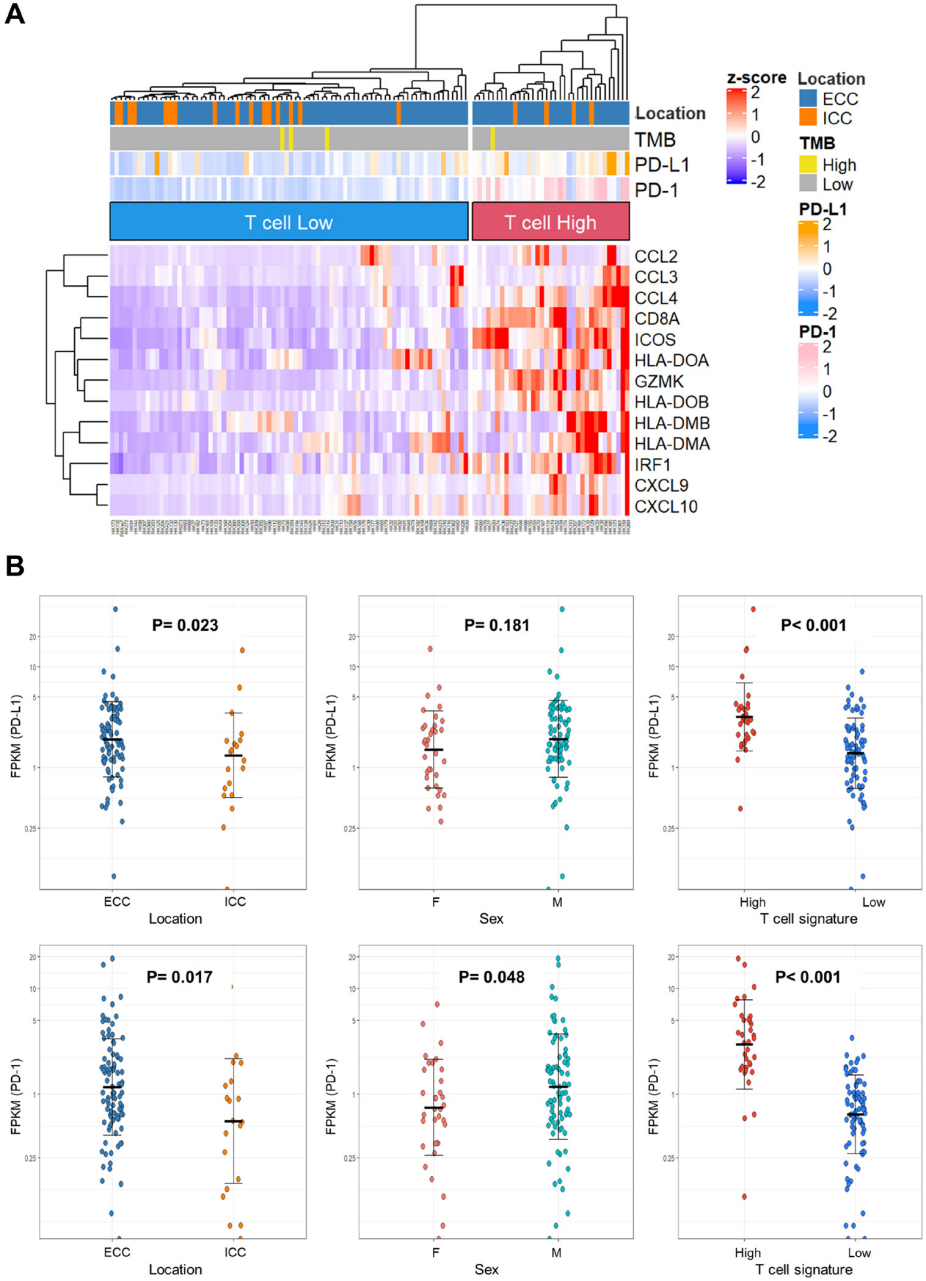
Immune-signature analysis of biliary tract cancer by RNA sequencing. (**A**) Heat map with 115 biliary tract cancers clustered to T-cell-High and -Low expression. (**B**) Dot plots of *PD-L1* and *PD-1* expression in clinicopathological data. The Y-axis is plotted on a logarithmic scale. Differences in gene expression were analyzed using the Mann–Whitney *U* test; *P* < 0.05 was considered statistically significant. Abbreviations: TMB: tumor mutation burden; ECC: extrahepatic cholangiocarcinoma; ICC: intrahepatic cholangiocarcinoma; FPKM: fragments per kilobase of exon per million reads mapped.

In addition to TMB, T cell expression and *PD-L1* and *PD-1* expression may be predictive biomarkers for ICIs. Here, we investigated the expression of predictive ICI biomarkers based on the RNA-seq results. The clinicopathological data of 115 cases with RNA-seq are shown in Supplementary Table 11. T-cell signature genes [23] were clustered by expression levels and categorized as T-cell-high and -low expression (Figure 3A). Thirty-five cases (30.4%) were classified as having T-cell-high expression. When BTC subgroups, TMB-High, *PD-L1*, and *PD-1* expression levels were annotated on the heat map, the T-cell-high group tended to have more ECC and higher *PD-L1* and *PD-1* expression.

Based on the report that high *PD-L1* expression was associated with pembrolizumab response [16], the relationship between *PD-L1* and *PD-1* expression and the location of BTC, sex, and T-cell expression were investigated (Figure 3B). *PD-L1* expression was found to be significantly higher in ECC and T-cell-high expression (*P* = 0.023 and *P* < 0.001, respectively). *PD-1* expression was significantly higher in ECC, males, and T-cell-high expression (*P* = 0.017, *P* = 0.048, and *P* < 0.001, respectively).

There were stronger associations between T cell hyper-expression and high *PDL-1* or *PD-1* expression. Assuming T-cell-high expression as an indicator, 35 cases had the actionability for ICI. Four patients had actionability for both molecular therapy and ICI. In total, 47.9% (105/219 cases) of BTC cases had actionability for molecular therapy or ICIs.

## DISCUSSION

In this study, 22 actionable genes and 43 candidate drugs were identified in 219 BTCs using the Asian KB. *PTEN* and *CDKN2A* were most frequently detected among the annotated actionable genes, and their mutations affected OS and RFS. We also characterized the biomarkers of ICIs, TMB, T cell expression, *PD-L1*, and *PD-1* in BTCs using RNA-seq to evaluate the immunological actionability for BTC.

Reports that estimated actionable genes using surgical specimens of BTC showed frequencies of 25% and 38.9%, respectively, [6, 24], which are similar to the frequencies found in this study (34.7%). In contrast, a report with a high frequency of actionable genes (54.8%) used surgical and biopsy specimens, including a high proportion of pathological stage IV (54.8%) [25]. Differences in the stage of malignancy progression may be one of the reasons for the different frequencies of actionable genes. Thus, the actionable genes annotated in this study may be interpreted as targets of postoperative adjuvant chemotherapy for BTC. A study using the same KB revealed the frequency of actionable genes in non-small cell lung cancer (68%), colorectal cancer (52%), breast cancer (52%), gastric cancer (28%), and small cell lung cancer (13%) [17]. In gastric cancer, which shows a frequency similar to that in this study, trastuzumab targeted at *ERBB2* and ramucirumab targeted at *VEGFR* have been observed to be effective for treatment [26]. Thus, development of drugs targeting actionable genes may also have some effect on BTC.

In this study, actionable genes in the MAPK pathway were observed to be the most frequent. *KRAS* mutation representative of the MAPK pathway is identified in 11–27% of BTC [6–8]. The MEK1/2 inhibitors trametinib [27] and selumetinib [28] have been evaluated in clinical trials for advanced BTC. Sotorasib, a new drug that directly targets *KRAS*, has been studied and has shown encouraging anticancer activity in phase I trials in patients with *KRAS p.G12C* mutation-positive advanced solid tumors [29].

In contrast, *PTEN* and *CDKN2A* were the most frequently identified actionable genes in this study. Notably, *PTEN* and *CDKN2A* were associated with worse OS and RFS. The prognostic effect of these variants has been reported previously [30], supporting this result. *PTEN*, a tumor suppressor gene, is a negative regulator of the PI3K/mTOR pathway, and abnormal activation of the PI3K/mTOR pathway has been associated with the development of malignancies, including BTC [31]. GSK2636771 [32] and AZD8186 [33], the PI3K inhibitors annotated in this study, are currently in the research phase of drug development. However, the efficacy of everolimus, an mTOR inhibitor, has also been studied, and positive results have been reported [34]. *CDKN2A* is also a tumor suppressor gene that encodes the CDK4/6 inhibitor p16ink4a [35]. Loss of this tumor suppressor gene contributes to the bypass of necessary aging signals and is associated with malignant disease progression. *CDKN2A* aberrations have been reported in 32% of ECCs and 28% of ICCs, making *CDKN2A* a new therapeutic target of interest [36, 37]. The CDK4/6 inhibitors abemaciclib, palbociclib, and ribociclib were annotated in this study. Further, palbociclib is reported to inhibit the growth of BTC cell lines *in vitro* [38].

Clinical trials of new drugs for BTC tend to focus on ICC. In a clinical trial of *IDH1* mutations (ClarIDHy) [10], 90% of patients with *IDH1* mutations had ICC. In a clinical trial of *FGFR2* mutations (FIGHT202) [11], 98% of patients with *FGFR2* fusion or rearrangements had ICC. These trials have demonstrated the clinical benefits of molecularly targeted drugs. In this study, *IDH1* was observed only in ICC. *FGFR2* was more frequently observed in ICC. Furthermore, the overall actionable genes were more frequently observed in ICC. The results of this study thus indicate that ICC may benefit more from molecular-targeted therapy.

This study also characterized the biomarkers of ICI and focused on *PD-L1* and *PD-1* expression. Fontugne et al. [39] reported an association between *PD-L1* expression and T cell infiltration in BTC, supporting the results of this study. Kriegsmann et al. showed that *PD-L1* expression in BTC cells was comparable between ICC (5%), PHC (4%), and DCC (3%), whereas *PD-L1* expression in stromal cells was the highest in DCC (61% in DCC, 40% in PHC, and 31% in ICC) [40]. Thus, high *PD-L1* expression in ECC in this study may be because of the high *PD-L1* expression in stromal cells. Pembrolizumab for head and neck squamous cell carcinoma has shown efficacy in patients with positive PD-L1 expression in cancer tissues, including stromal cells [41]. Thus, pembrolizumab may be useful in ECC as well.

This study had some limitations. First, the analysis was performed on surgical specimens and may not reflect actionable genes in advanced BTC. Second, the sequencing method was not uniform in this study. The TMB-High thresholds were set separately, but there were differences in the frequency of TMB-High between WGS/WES and targeted sequencing, which may have led to misclassification. A uniform sequencing method should be used to re-evaluate TMB. Finally, this cohort did not receive any of the candidate drugs annotated in this study; therefore, the usefulness of candidate drugs needs to be assessed.

However, due to the rarity of BTC, large-scale clinical trials are challenging to establish. Recently, it was demonstrated that BTC cell lines and organoids have genomic alterations similar to those in primary tumors, indicating that they could be useful in developing and validating therapeutic targets for BTC [42]. As shown in this study, identifying actionable genes and candidate drugs using KBs could facilitate comprehensive validation using cell lines and organoids. This may further contribute to the development of therapeutic medications for BTC.

In conclusion, we identified 22 actionable genes and 43 candidate drugs for BTC using a KB with research-level information on genomic alterations. We also characterized the biomarkers of ICIs using RNA-seq. Further validation of comprehensive candidate drugs using cell lines and organoids based on these data, may facilitate drug discovery for BTC.

## MATERIALS AND METHODS

### Patients

Two hundred and nineteen patients diagnosed with BTC were enrolled at Hokkaido University Gastroenterological Surgery II between 2003 and 2018. Two hundred and eighteen patients with BTC were enrolled in the International Cancer Genome Consortium (ICGC) (Fujimoto et al. 2015 [20], 2016 [21], Wardell 2018 [19]), and these data were included. One case of gallbladder carcinoma (RK560) was then added and sequencing data were analyzed. For all cases, fresh frozen tumors and normal tissues were obtained during surgery. All clinical data were collected from electronic medical records, and the median observation period was 34 months. The pathological diagnosis was based on the AJCC/UICC 7th edition. The clinic-pathological data of the patients are shown in Supplementary Table 1. Of 219 cases, Whole-genome sequencing (WGS) was performed in 40 cases, whole-exome sequencing (WES) in 107 cases, and targeted sequencing in 72 cases. RNA sequencing (RNA-seq) was performed in 115 of 219 cases. Written informed consent was obtained from all patients in this study, following ICGC guidelines. In compliance with the Declaration of Helsinki, this study was approved by the institutional review boards at RIKEN (H20-16), Hokkaido University (16–051), and all participating institutions.

### Library preparation

Exome capture was performed using the Nextera Rapid Capture Exomes kits (Illumina) for all 107 cases that underwent WES. For WGS, DNA was extracted from the tumor and normal tissue, and 500–600-bp insert libraries were prepared following the manufacturer’s protocol. The exome-capture or WGS libraries were sequenced on HiSeq2000/2500 with paired reads of 100–125 bp. Targeted sequencing was performed on Illumina HiSeq2000/2500 after capturing using the SureSelect XT Custom kit (Agilent Technologies) following the manufacturer’s protocol. RNA-seq libraries were prepared using the TruSeq Stranded mRNA Sample Prep kit (Illumina) or the KAPA RNA HyperPrep Kit with RiboErase (Roche) following the manufacturer’s protocol. The Truseq Stranded mRNA Sample Prep Kit was used in 87 cases, and the KAPA RNA HyperPrep Kit with RiboErase was used in the other 28 cases. RNA-seq was performed on the HiSeq2500 platform.

### Mutation calls, CNV calls, and RNA-seq analysis

Single nucleotide variants (SNVs) and indels were detected using the in-house pipeline Genomon2 (https://genomon.readthedocs.io/ja/v2.6.1/). Briefly, sequence reads were mapped to the human reference genome GRCh37 using BWA [43], and the resulting files were converted to a pipe-up file using samtools [44]. PCR duplicates were removed using Picard (http://broadinstitute.github.io/picard), and after comparing the results from cancerous and normal tissues, SNVs and indels were detected using Genomon2. Copy number variation (CNV) in WGS and WES was calculated using Sequenza R [45], and genome sequencing data from pairs of normal-tumor samples were analyzed. GISTIC 2.0, was used to identify CNVs [46]. RNA-Seq reads were mapped to the human reference genome GRCh37, using STAR [47]. Gene fusions were detected using fusionfusion (https://github.com/Genomon-Project/fusionfusion).

### Annotation of actionable genes and candidate drugs

CancerSCAN^®^ [18] is a platform for cancer genome analysis, covering all the algorithms for sequencing data analysis and data interpretation. The annotation KB included in CancerSCAN^®^ is a real-world database containing information matching cancer-related genes and mutations curated by the Samsung Genome Institute and has a high frequency of East Asians (15,000 Koreans) in particular. Using this database, tier information that maps drugs to tumor types can be obtained based on the ACMG guidelines. In addition, the KB can be used throughout cancer genome analysis, such as for predicting the actionability for variants of unknown significance (VUS) through variant frequency information.

### Gene categorization

The actionable genes found to be altered in this study were classified in terms of the pathways involved based on the literature as follows [37, 48]: mitogen-activated protein kinase (MAPK), *BRAF, HRAS, KRAS,* and *NF1*; receptor tyrosine kinase (RTK), *ALK, EGFR, ERBB2, FGFR1, FGFR2, FGFR3, FLT3,* and *MET*; phosphoinositide 3-kinase (PI3K)/mammalian target of rapamycin (mTOR) signaling, *PIK3CA, PTEN,* and *TSC1*; cell cycle control, *CDKN2A* and *CDK4*; DNA damage repair, *ATM, BRCA1,* and *BRCA2*; p53 signaling pathway, *MDM2*; and metabolic pathway, *IDH1*.

### Definition of tumor mutation burden

TMB is defined as the number of nonsynonymous somatic mutations, coding mutations, base substitution mutations, and indel mutations per megabase (Mb) of the genome. When calculating the TMB, we divided the total number of mutations by 39 Mbp for WGS and WES and by 0.196 Mbp for targeted sequencing. There are still no clear criteria for the cut-off value for TMB-High. The cut-off value for TMB-High of pembrolizumab for solid tumors, approved by the Food and Drug Administration (FDA) in 2020, was 10 mutations/Mbp [12]; in the WGS and WES in this study, TMB ≥ 10 mutations/Mbp was defined as TMB-High. However, Budczies et al. [49] described that the misclassification rate of TMB-High increased with the decreasing coding sequence region in panel sequencing. They reported that in a panel with a coding sequence region of 0.21 Mbp, defining TMB-High as ≥ 4/Mbp reduced misclassification. Based on this report, TMB ≥ 20.4 mutations (4 mutations/sample) was defined as TMB-High for target sequencing in this study.

### Immune-signature analysis

To predict the effect of ICIs, the expression levels of T cell signature genes [23] (*CD8A, CCL2, CCL3, CCL4, CXCL9, CXCL10, ICOS, GZMK, IRF1, HLA-DMA, HLA-DMB, HLA-DOA,* and *HLA-DOB*), *PD-L1* (*CD274*), and *PD-1* (*CD279*) in 115 patients who underwent RNA-seq were determined. RNA-seq reads were mapped to the reference sequence using STAR [50]. Duplicate reads were identified using Picard MarkDuplicates and BAM files were generated. The number of reads was calculated using featureCounts [51] and the results were normalized as Fragments per kilobase of exons per million reads (FPKM). The expression levels of 13 T-cell signature genes were clustered using Ward’s method. A heat map was created using the ComplexHeatmap R package [52].

### Statistical analysis

All calculations were performed using R software (version 3.6.3). Continuous variables are expressed as median, minimum, and maximum. Nominal variables are expressed as frequencies and percentages. Continuous variables were analyzed using Mann–Whitney *U* test, and nominal variables were analyzed using chi-square test or Fisher’s exact test. Survival curves were plotted using the Kaplan–Meier method and were compared using the log-rank test. Statistical significance was set at *p* < 0.05.

## SUPPLEMENTARY MATERIALS





## Abbreviations

BTC: biliary tract cancer
CDC: cystic ductal carcinoma
CNV: copy number variation
DCC: distal cholangiocarcinoma
ESMO: European society for medical ontology
FPKM: Fragments per kilobase of exons per million reads
GBC: gallbladder carcinoma
ICGC: International Cancer Genome Consortium
ICI: immune checkpoint inhibitor
ICC: intrahepatic cholangiocarcinoma
KB: knowledge base
MSI: microsatellite instability
OS: overall survival
PHC: perihilar cholangiocarcinoma
RFS: relapse-free survival
RNA-seq: RNA sequencing
SNV: single nucleotide variant
TMB: tumor mutation burden
