# Supplementary material for: Actionability evaluation of biliary tract cancer by genome transcriptome analysis and Asian cancer knowledgebase
Source: Oncotarget. 2021 Jul 20;12(15):1540–52. doi: 10.18632/oncotarget.28021 (PMC8310666; doi:10.18632/oncotarget.28021)
Supplement: Supplementary file 1 [file oncotarget-12-1540-s001.pdf]

Actionability evaluation of biliary tract cancer by genome transcriptome analysis and Asian cancer knowledgebase

SUPPLEMENTARY MATERIALS

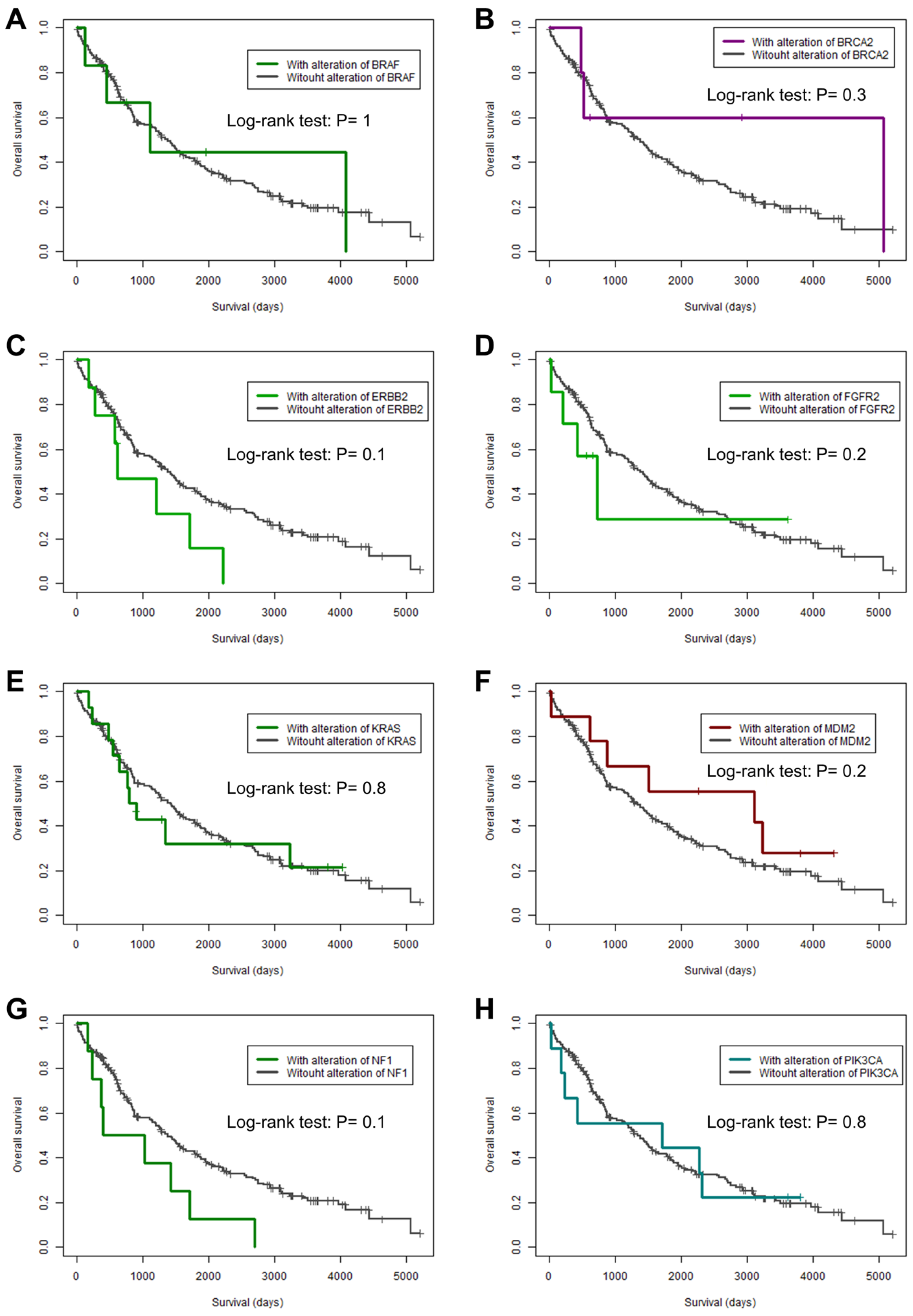

Supplementary Figure 1: Kaplan–Meier curves for overall survival in actionable genes. (**A**) *BRAF*, (**B**) *BRCA2*, (**C**) *ERBB2*, (**D**) *FGFR2*, (**E**) *KRAS*, (**F**) *MDM2*, (**G**) *NF1*, (**H**) *PIK3CA*. The differences in overall survival and relapse-free survival were analyzed by log-rank test; $P < 0.05$ was considered statistically significant.

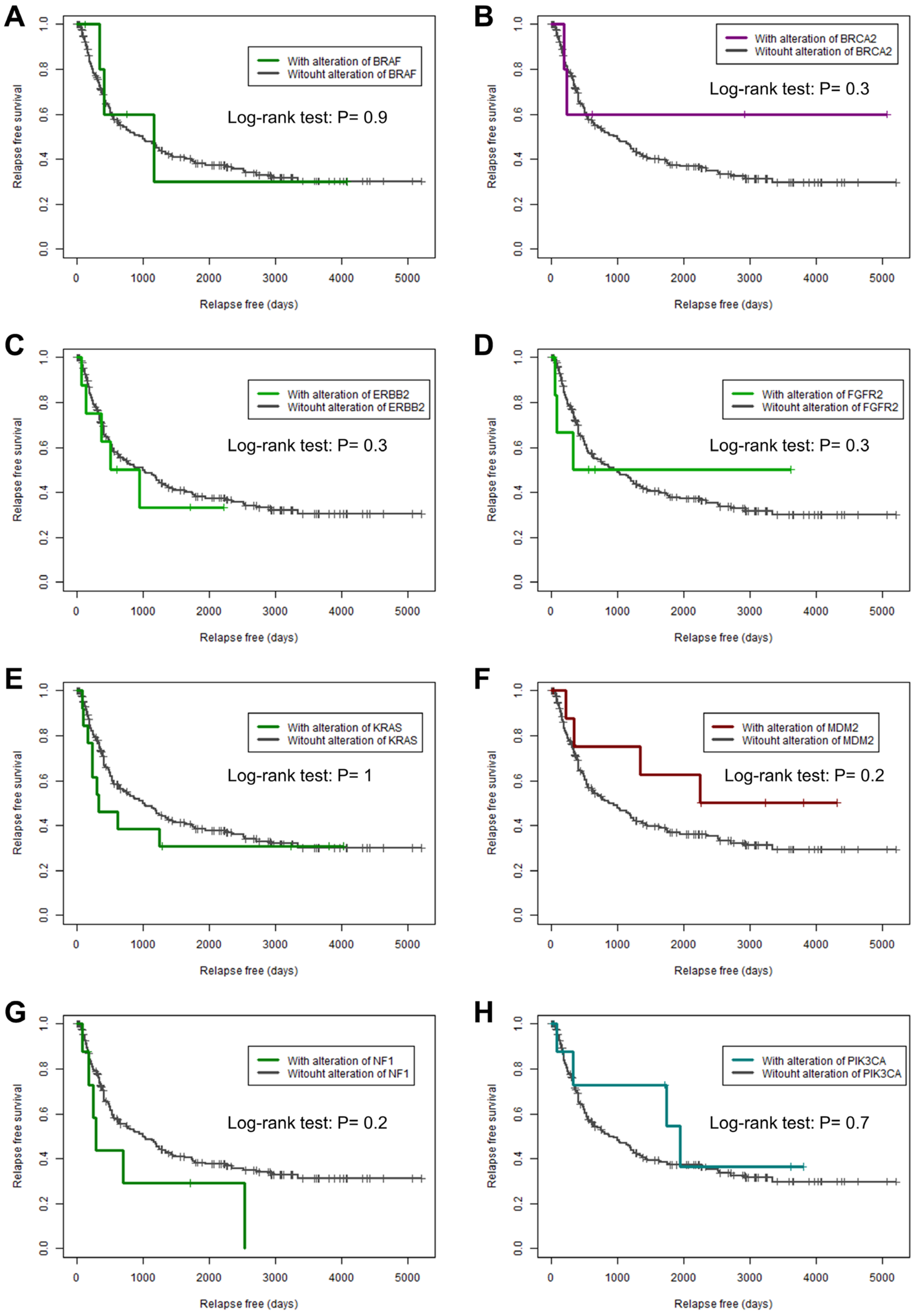

Supplementary Figure 2: Kaplan–Meier curves for relapse-free survival in actionable genes. (**A**) *BRAF*, (**B**) *BRCA2*, (**C**) *ERBB2*, (**D**) *FGFR2*, (**E**) *KRAS*, (**F**) *MDM2*, (**G**) *NF1*, (**H**) *PIK3CA*. The differences in overall survival and relapse-free survival were analyzed by log-rank test; $P < 0.05$ was considered statistically significant.

Supplementary Table 1: Patient clinical data of 218 biliary tract cancer

ID	Sequencing method	Subtype	Gender	Age	Histology	pT	pN	pM	pStage	OS (days)	Event of death	RFS (days)	Event of relapse
RK073	WGS	ICC	M	62	mod	1	0	0	1	385	(–)	160	(+)
RK109	WGS	ICC	M	84	cholangiocellular carcinoma	2	0	0	2	930	(–)	155	(+)
RK137	WGS	ICC	F	74	mod	3	0	0	3	920	(–)	570	(+)
RK138	WGS	ICC	F	75	por	3	0	0	2	242	(+)	92	(+)
RK142	WGS	ICC	M	57	well	1	0	0	1	625	(–)	625	(–)
RK146	WGS	ICC	F	57	mod	2	0	0	2	902	(–)	310	(+)
RK182	WGS	ICC	M	65	mod	3	0	0	3	21	(+)	21	(–)
RK194	WGS	ICC	M	67	well	3	0	0	3	1290	(–)	1290	(–)
RK204	WGS	ICC	M	83	well	1	0	0	1	755	(–)	755	(–)
RK208	WGS	ICC	M	60	mod	3	0	0	3	17	(–)	17	(–)
RK226	WGS	ICC	M	59	mod	3	0	0	3	480	(–)	358	(+)
RK269	WGS	ICC	M	74	well	1	0	0	1	399	(–)	399	(–)
RK272	WGS	ICC	M	78	mod	2	0	0	2	370	(–)	370	(–)
RK279	WGS	ICC	M	69	mod	3	0	0	3	560	(–)	560	(–)
RK298	WGS	ICC	M	68	por	3	0	0	3	440	(–)	440	(–)
RK303	WGS	ICC	M	76	cholangiocellular carcinoma	2	0	0	2	285	(–)	285	(–)
RK307	WGS	ICC	F	61	mod	4	1	0	4	1286	(+)	500	(+)
RK308	WGS	ICC	F	70	mod	3	0	0	3	3624	(–)	3624	(–)
RK309	WGS	ICC	M	56	mod	4	1	0	4	526	(+)	193	(+)
RK310	WGS	ICC	F	62	mod	3	0	0	3	1782	(+)	321	(+)
RK312	WGS	ICC	M	66	mod	3	1	0	4	525	(–)	130	(+)
RK316	WGS	ICC	F	54	mod	3	3	0	4	240	(+)	90	(+)
RK317	WGS	ICC	M	73	mod	3	0	0	3	28	(+)	28	(–)
RK353	WGS	CDC	F	71	well	1	0	0	1	2235	(–)	2235	(–)
RK354	WGS	CDC	M	61	mod	3	1	0	4	25	(+)	25	(–)
RK355	WGS	CDC	M	68	mod	3	0	0	3	651	(+)	189	(+)
RK356	WGS	CDC	F	84	mod	3	1	0	4	4093	(+)	4093	(–)
RK357	WGS	GBC	M	82	well	2	0	0	2	2228	(+)	2228	(–)
RK358	WGS	CDC	M	76	mod	2	0	0	2	3420	(–)	3420	(–)
RK359	WGS	GBC	F	86	mod	3	3	0	4	277	(+)	134	(+)
RK360	WGS	GBC	F	82	well	1	0	0	1	1718	(+)	1718	(–)
RK361	WGS	GBC	M	63	adeno-endocrine	3	1	0	4	648	(+)	536	(+)
RK362	WGS	GBC	F	61	well	2	0	0	2	3073	(–)	3073	(–)
RK419	WGS	DCC	F	71	mod	3	0	0	3	4078	(–)	4078	(–)
RK420	WGS	DCC	M	67	mod	3	1	0	4	540	(+)	173	(+)
RK421	WGS	DCC	F	77	por	1	0	0	1	1614	(–)	1614	(–)
RK422	WGS	DCC	M	70	por	2	0	0	2	2552	(+)	2552	(–)
RK423	WGS	DCC	M	75	muc	2	1	0	3	397	(+)	183	(+)
RK424	WGS	DCC	M	69	well	2	1	0	3	409	(+)	126	(+)
RK560	WGS	GBC	M	51	mod	2	0	0	2	613	(–)	613	(–)

Supplemetnary Table 2: The gene list of genetic analysis using genomic DNA

ABCB1	BACH1	CDH1	DPYD	FAM175	FGFR3	HSPH1	LRP6	NBN	PIK3CB	RET	SRC	WEE1
ABCC1	BAP1	CDH2	EGF	FANCA	FGFR4	IDH1	LTK	NCOA3	PIK3CD	RHBDF2	SRSF2	WT1
ABCC3	BARD1	CDH5	EGFR	FANCC	FLCN	IDH2	MAD1L1	NCOR1	PIK3CG	RICTOR	STAG2	XPA
ABCG2	BCL2	CDK12	EML4	FANCD2	FLI1	IDO1	MAP2K1	NF1	PIK3R1	RIT1	STAT3	XPC
ABL1	BCL2A1	CDK4	EP300	FANCE	FLT1	IGF1	MAP2K2	NF2	PIK3R2	RNF43	STAT4	XRCC1
ABL2	BCL2L1	CDK6	EPHA2	FANCF	FLT3	IGF1R	MAP2K4	NFKBIA	PKHD1	ROS1	STK11	XRCC2
AKT1	BCL2L2	CDK8	EPHA3	FANCG	FLT4	IGF2	MAP2K7	NKX2-1	PLCG1	RPS6KB	SUZ12	XRCC3
AKT2	BCL6	CDKN1A	EPHA6	FANCI	FOXA1	IGF2R	MAP3K1	NOTCH	PML	RPTOR	SYK	ZNF217
AKT3	BCOR	CDKN1B	EPHA7	FANCL	FOXL2	IKBKE	MAPK1	NPM1	PMS2	RRAS2	TBX3	ZNRF3
ALK	BCORL1	CDKN2	EPHB1	FANCM	FOXP1	IKZF1	MCL1	NRAS	POLE	RSF1	TERT	
APC	BIRC7	CDKN2A	EPHB4	FAT1	FRS2	IL1B	MDM2	NRG1	PPARG	RUNX1	TET2	
APCDD1	BLM	CDKN2B	EPHB6	FAT3	G6PD	IL7R	MDM4	NSD1	PPP1R1	SDHAF2	TFRC	
APOBEC3	BRAF	CDX2	ERBB2	FBXW7	GATA1	INPP4B	MED12	NT5C2	PPP2R2	SDHB	TGFBR1	
AR	BRCA1	CEBPA	ERBB3	FCGR2B	GATA2	INSR	MEN1	NTRK1	PRDM1	SDHC	TGFBR2	
ARAF	BRCA2	CHD4	ERBB4	FGF1	GATA3	IRS2	MET	NTRK2	PTCH1	SDHD	TMPRS	
AREG	BRIP1	CHEK1	ERCC1	FGF10	GEN1	ITK	MITF	NTRK3	PTCH2	SERPIN	TNFAIP3	
ARID1A	CALR	CHEK2	ERCC2	FGF14	GLI2	JAK1	MLH1	PAK1	PTEN	SETBP1	TNFRSF	
ARID1B	CARD11	CHUK	ERCC3	FGF18	GNA11	JAK2	MLLT3	PAK7	PTPN11	SETD2	TNKS	
ARID2	CASP8	CIC	ERCC4	FGF19	GNAQ	JAK3	MPL	PALB2	PTPRD	SF3B1	TOP1	
ASNS	CBFB	CREBB	ERCC5	FGF2	GNAS	KAT6A	MRE11A	PARP1	RAB35	SH2B3	TOP2A	
ASXL1	CBL	CSF1R	ERCC6	FGF23	GSTP1	KCNJ5	MSH2	PARP2	RAC1	SLC29A	TP53	
ATM	CBLB	CSF3R	ERG	FGF3	H19	KDM5A	MSH3	PARP3	RAD21	SLX4	TPMT	
ATR	CCND1	CTNNB1	ESR1	FGF4	H3F3A	KDR	MSH6	PARP4	RAD50	SMAD2	TSC1	
ATRX	CCND2	CUL4A	ETS1	FGF5	HDAC2	KIF5B	MTHFR	PAX3	RAD51	SMAD4	TSHR	
AURKA	CCND3	CYP17A	ETV1	FGF6	HGF	KIT	MTOR	PAX7	RAD51B	SMARC	TTF1	
AURKB	CCNE1	DDR2	ETV4	FGF7	HIF1A	KMT2A	MUTYH	PBRM1	RAD51C	SMO	TYMS	
AXIN1	CD274	DIS3	ETV5	FGF8	HIST1H	KMT2D	MYC	PDCD1L	RAD51D	SOCS1	U2AF1	
AXIN2	CD79A	DNMT1	ETV6	FGF9	HNF1A	KRAS	MYCL1	PDGFR	RAD54L	SOX2	UGT1A1	
AXL	CD79B	DNMT3	EWSR1	FGFR1	HRAS	LAMP1	MYCN	PDPK1	RAF1	SPEN	VEGFA	
B2M	CDC73	DOT1L	EZH2	FGFR2	HSP90A	LRP1B	MYD88	PIK3CA	RB1	SPOP	VHL	

Supplementary Table 3: The gene list of transcriptome profiling using RNA. See Supplementary Table 3

Supplementary Table 4: Actionable genes in single nucleotide variants and indels

Gene	**Patients (*n* = 219)**		**No of variants**	**Amino acid substitutions by oncogenic variants**	**Candidate drugs**
KRAS	11	(5 %)	11	Gly12Asp, Gly12Val, Gly12Ala, Gly12Cys, Ala146Thr	Cobimetinib, Binimetinib Trametinib
NF1	7	(3.2 %)	8	Gln543X, Gln1763X, Arg1241X, Glu1789X, Arg2517X, Gln1235X, Gln1399X, Arg2450X	Cobimetinib, Trametinib
CDKN2A	5	(2.3 %)	5	Asp84Asn, His83Tyr, Arg80X, Ala73fs, Asp84Asn	Abemaciclib, Palbociclib Ribociclib
PIK3CA	5	(2.3 %)	5	Glu453Lys, Glu545Lys	Alpelisib + Fulvestrant, Buparlisib Serabelisib, Copanlisib, GDC-0077 Taselisib + Fulvestrant, Alpelisib, Buparlisib + Fulvestrant, Taselisib
BRAF	4	(1.8 %)	4	Gly466Val, Asp594Asn, Gly469Ala, Lys601Asn	Cobimetinib, PLX8394
ERBB2	3	(1.4 %)	3	Arg678Gln, Leu755Ser, Ser310Phe	Ado-Trastuzumab Emtansine Neratinib, Neratinib
ATM	2	(0.9 %)	2	Tyr729X, Gln1084X	Olaparib
IDH1	2	(0.9 %)	2	Arg132Ser, Arg132Gly	Ivosidenib
BRCA1	1	(0.5 %)	1	Glu1107X	Rucaparib, Niraparib Talazoparib, Olaparib
BRCA2	1	(0.5 %)	1	Ser2095X	Rucaparib, Niraparib Talazoparib, Olaparib

Data are expressed as number (%).

Supplementary Table 5: Actionable genes in copy number variants

Gene	Alteration	Patients (n = 219)		Candidate drugs
PTEN	Loss	16	(7.3 %)	GSK2636771, AZD8186
CDKN2A	Loss	10	(4.6 %)	Abemaciclib, Palbociclib, Ribociclib
MDM2	Gain	9	(4.1 %)	Milademetan Tosylate, RO5045337
ERBB2	Gain	5	(2.3 %)	Lapatinib + Trastuzumab, Pertuzumab + Trastuzumab Ado-Trastuzumab Emtansine, Lapatinib Neratinib, Trastuzumab
CDK4	Gain	4	(1.8 %)	Abemaciclib, Palbociclib
FGFR3	Gain	4	(1.8 %)	AZD4547, Erdafitinib, BGJ398, Debio1347
PIK3CA	Gain	4	(1.8 %)	Alpelisib + Fulvestrant
ALK	Gain	3	(1.4 %)	Brigatinib, Lorlatinib
FGFR1	Gain	3	(1.4 %)	AZD4547, Erdafitinib, BGJ398, Debio1347
FGFR2	Gain	3	(1.4 %)	AZD4547, Erdafitinib, BGJ398, Debio1347
KRAS	Gain	3	(1.4 %)	Cobimetinib, Binimetinib, Trametinib
MET	Gain	3	(1.4 %)	Crizotinib
BRAF	Gain	2	(0.9 %)	Cobimetinib
BRCA2	Loss	2	(0.9 %)	Rucaparib, Niraparib
EGFR	Gain	2	(0.9 %)	Lapatinib
FLT3	Gain	2	(0.9 %)	Midostaurin + High Dose Chemotherapy
BRCA1	Loss	1	(0.5 %)	Rucaparib, Niraparib
HRAS	Gain	1	(0.5 %)	Tipifarnib
NF1	Loss	1	(0.5 %)	Cobimetinib, Trametinib
TSC1	Loss	1	(0.5 %)	Everolimus

Data are expressed as number (%).

Supplementary Table 6: Actionable genes in fusions

Gene	Patients (n = 219)		No. of fusion	Candidate drugs
CASP7-FGFR2	2	(0.9 %)	4	Erdafitinib
ERC1-FGFR1	1	(0.5 %)	1	AZD4547, BGJ398, Erdafitinib, Debio1347
VCL-FGFR2	1	(0.5 %)	3	Erdafitinib
BICC1-FGFR2	1	(0.5 %)	3	AZD4547, Erdafitinib, BGJ398, Debio1347
PLEKHS1-FGFR2	1	(0.5 %)	3	Erdafitinib
ABLIM1-FGFR2	1	(0.5 %)	1	Erdafitinib

Data are expressed as number (%).

Supplementary Table 7: Candidate drugs annotated in patients with biliary tract cancer

Candidate drugs	Actionable gene	Patients (n = 219)	
Cobimetinib	*BRAF, KRAS, NF1*	25	(11.4 %)
Trametinib	*NF1*	19	(8.7 %)
Abemaciclib	*CDK4, CDK2A*	18	(8.2 %)
Palbociclib	*CDK4, CDKN2A*	18	(8.2 %)
AZD8186	*PTEN*	16	(7.3 %)
GSK2636771	*PTEN*	16	(7.3 %)
Erdafitinib	*FGFR1/2/3*	15	(6.8 %)
Ribociclib	*CDKN2A*	15	(6.8 %)
Cetuximab	*KRAS*	14	(6.4 %)
Panitumumab	*KRAS*	14	(6.4 %)
AZD4547	*FGFR1/2/3*	12	(5.5 %)
BGJ398	*FGFR1/2/3*	12	(5.5 %)
Debio1347	*FGFR1/2/3*	12	(5.5 %)
Binimetinib	*KRAS*	11	(5 %)
Alpelisib + Fulvestrant	*PIK3CA*	9	(4.1 %)
Milademetan Tosylate	*MDM2*	9	(4.1 %)
RO5045337	*MDM2*	9	(4.1 %)
Ado-Trastuzumab Emtansine	*ERBB2*	8	(3.7 %)
Neratinib	*ERBB2*	8	(3.7 %)
Niraparib	*BRCA1/2*	8	(3.7 %)
Rucaparib	*BRCA1/2*	8	(3.7 %)
Lapatinib	*ERBB2, EGFR*	7	(3.2 %)
Olaparib	*ATM, BRCA1/2*	7	(3.2 %)
Alpelisib	*PIK3CA*	5	(2.3 %)
Buparlisib	*PIK3CA*	5	(2.3 %)
Buparlisib + Fulvestrant	*PIK3CA*	5	(2.3 %)
Copanlisib	*PIK3CA*	5	(2.3 %)
GDC-0077	*PIK3CA*	5	(2.3 %)
Lapatinib + Trastuzumab	*ERBB2*	5	(2.3 %)
Pertuzumab + Trastuzumab	*ERBB2*	5	(2.3 %)
Serabelisib	*PIK3CA*	5	(2.3 %)
Talazoparib	*BRCA1/2*	5	(2.3 %)
Taselisib	*PIK3CA*	5	(2.3 %)
Taselisib + Fulvestrant	*PIK3CA*	5	(2.3 %)
Trastuzumab	*ERBB2*	5	(2.3 %)
Brigatinib	*ALK*	3	(1.4 %)
Crizotinib	*MET*	3	(1.4 %)
Lorlatinib	*ALK*	3	(1.4 %)
Ivosidenib	*IDH1*	2	(0.9 %)
Midostaurin + High Dose Chemotherapy	*FLT3*	2	(0.9 %)
Everolimus	*TSC1*	1	(0.5 %)
PLX8394	*BRAF*	1	(0.5 %)
Tipifarnib	*HRAS*	1	(0.5 %)

Data are expressed as number (%).

Supplementary Table 8: Comparison between actionable genes and sex

	Male *n* = 159		Female *n* = 60		*P*-value	OR	95% CI
Actionability (+)	50	(31.4)	24	(40)	0.3	0.69	(0.37–1.27)
ALK	1	(0.6)	2	(3.3)	0.18	0.19	(0–3.62)
ATM	2	(1.3)	0	(0)	1	Inf	#VALUE!
BRAF	3	(1.9)	3	(5)	0.35	0.37	(0.05–2.82)
BRCA1	1	(0.6)	1	(1.7)	0.47	0.38	(0–29.8)
BRCA2	3	(1.9)	0	(0)	0.56	Inf	(0.16-Inf)
CDK4	1	(0.6)	3	(5)	0.06	0.12	(0–1.55)
CDKN2A	10	(6.3)	5	(8.3)	0.56	0.74	(0.22–2.88)
EGFR	2	(1.3)	0	(0)	1	Inf	(0.07-Inf)
ERBB2	4	(2.5)	4	(6.7)	0.22	0.36	(0.07–2.02)
FGFR1	2	(1.3)	2	(3.3)	0.3	0.37	(0.03–5.23)
FGFR2	6	(3.8)	1	(1.7)	0.68	2.31	(0.27–108.16)
FGFR3	3	(1.9)	1	(1.7)	1	1.13	(0.09–60.54)
FLT3	2	(1.3)	0	(0)	1	Inf	(0.07-Inf)
HRAS	1	(0.6)	0	(0)	1	Inf	(0.01-Inf)
IDH1	1	(0.6)	1	(1.7)	0.47	0.38	(0–29.8)
KRAS	8	(5)	6	(10)	0.22	0.48	(0.14–1.75)
MDM2	5	(3.1)	4	(6.7)	0.26	0.46	(0.09–2.38)
MET	2	(1.3)	1	(1.7)	1	0.75	(0.04–45.08)
NF1	5	(3.1)	3	(5)	0.69	0.62	(0.12–4.11)
PIK3CA	6	(3.8)	3	(5)	0.71	0.75	(0.15–4.76)
PTEN	14	(8.8)	2	(3.3)	0.25	2.79	(0.61–26.06)
TSC1	1	(0.6)	0	(0)	1	Inf	(0.01-Inf)

Data are expressed as number (%). Statistical tests were performed using the chi-square test for the presence of Actionability and the Fisher test for each gene; $P < 0.05$ is considered statistically significant. Abbreviations: OR: odd ratio; CI: confidential interval.

Supplementary Table 9: Comparison between actionable genes and lymph node metastasis

	Lymph metastasis (+) *n* = 77		Lymph metastasis (–) *n* = 138		*P*-value	OR	95% CI
Actionability (+)	27	(35.1)	46	(33.3)	0.91	1.08	(0.6–1.94)
ALK	0	(0)	3	(2.2)	0.55	0	(0–4.34)
ATM	0	(0)	2	(1.4)	0.54	0	(0–9.55)
BRAF	2	(2.6)	4	(2.9)	1	0.89	(0.08–6.41)
BRCA1	0	(0)	2	(1.4)	0.54	0	(0–9.55)
BRCA2	1	(1.3)	2	(1.4)	1	0.01	(17.46–0.9)
CDK4	0	(0)	4	(2.9)	0.3	0	(0–2.71)
CDKN2A	7	(9.1)	7	(5.1)	0.26	1.87	(0.53–6.51)
EGFR	1	(1.3)	1	(0.7)	1	1.8	(0.02–142.36)
ERBB2	3	(3.9)	5	(3.6)	1	1.08	(0.16–5.72)
FGFR1	1	(1.3)	3	(2.2)	1	0.59	(0.01–7.54)
FGFR2	2	(2.6)	5	(3.6)	1	0.71	(0.07–4.47)
FGFR3	0	(0)	4	(2.9)	0.3	0	(0–2.71)
FLT3	0	(0)	2	(1.4)	0.54	0	(0–9.55)
HRAS	1	(1.3)	0	(0)	0.36	Inf	(0.05-Inf)
IDH1	0	(0)	2	(1.4)	0.54	0	(0–9.55)
KRAS	4	(5.2)	10	(7.2)	0.77	0.7	(0.16–2.55)
MDM2	2	(2.6)	7	(5.1)	0.49	0.5	(0.05–2.72)
MET	0	(0)	3	(2.2)	0.55	0	(0–4.34)
NF1	2	(2.6)	6	(4.3)	0.71	0.59	(0.06–3.39)
PIK3CA	2	(2.6)	7	(5.1)	0.49	0.5	(0.05–2.72)
PTEN	6	(7.8)	10	(7.2)	1	1.08	(0.31–3.45)
TSC1	0	(0)	1	(0.7)	1	0	(0–69.82)

Data are expressed as number (%). Statistical tests were performed using the chi-square test for the presence of Actionability and the Fisher test for each gene; $P < 0.05$ is considered statistically significant. Abbreviations: OR: odd ratio; CI: confidential interval.

Supplementary Table 10: Characteristics of patients with TMB ≥ 20.4 mutations/Mbp for targeted sequencing

ID	Subtype	Sex	Age	Number of SNV	Number of Indel	TMB	Actionable genes
RK428	ICC	M	77	19	0	96.9	None
RK426	ICC	F	63	18	0	91.8	*BRAF, BRCA2*
RK368	ICC	M	68	9	0	45.9	*BRCA2, KRAS*
HK130	PHC	F	70	4	2	30.6	None
PAXno6	GBC	F	69	6	0	30.6	None
HK112	DCC	F	NA	6	0	30.6	None
PAXno9	DCC	M	79	6	0	30.6	None
HK134	PHC	F	73	4	1	25.5	None
HK156	PHC	F	77	4	1	25.5	*NF1*
PAXno14	DCC	M	66	4	1	25.5	None
RK370	ICC	M	77	3	2	25.5	*FGFR2*
HK150	PHC	F	49	4	0	20.4	*BRAF*
PAXno4	PHC	M	66	4	0	20.4	*KRAS*
HK137	DCC	M	70	4	0	20.4	*KRAS*
HK146	DCC	F	81	4	0	20.4	*KRAS*
PAXno15	DCC	M	71	4	0	20.4	*CDKN2A*
RK306	ICC	M	54	3	1	20.4	*CDKN2A*
RK427	ICC	F	78	3	1	20.4	None

Abbreviations: TMB: tumor mutation burden; ICC: intrahepatic cholangiocarcinoma; PHC: perihilar cholangiocarcinoma; CDC: cystic duct carcinoma; GBC: gallbladder carcinoma; DCC: distal cholangiocarcinoma.

Supplementary Table 11: Characteristics of patients with biliary tract cancer for RNA sequencing

	RNA sequencing (*n* = 115)	
Age, years		
Median (range)	70	(47–86)
Sex		
Male	81	(70.4)
Female	34	(29.6)
The location of BTC		
ICC	20	(17.4)
PHC	27	(23.5)
CDC/ GBC	36	(31.3)
DCC	32	(27.8)
Pathological lymph node metastasis		
(–)	69	(60)
(+)	44	(38.3)
NA	2	(1.7)
Pathological Stage acording to AJCC/UICC 7th		
I	18	(15.7)
II	35	(30.4)
III	41	(35.7)
IV	20	(17.4)
NA	1	(0.9)
Histology		
Tubular adenocarcinoma	94	(81.7)
Poorly differented adenocarcinoma	14	(12.2)
Adenosquamous adenocarcinoma	2	(1.7)
Mucinous adenocarcinoma	1	(0.9)
Others	1	(0.9)
NA	3	(2.6)

Data are expressed as number (%) or median (minimum value – maximum value). Abbreviations: BTC: bile duct carcinoma; ICC: intrahepatic cholangiocarcainoma; PHC: perihilar cholangiocarcinoma; CDC: cystic duct carcinoma; GBC: gallbladder carcinoma; DCC: distal cholangiocarcinoma; NA: not available.
